# An Evaluation of Non-Contact Photoplethysmography-Based Methods for Remote Respiratory Rate Estimation

**DOI:** 10.3390/s23073387

**Published:** 2023-03-23

**Authors:** Giuseppe Boccignone, Alessandro D’Amelio, Omar Ghezzi, Giuliano Grossi, Raffaella Lanzarotti

**Affiliations:** PHuSe Laboratory—Dipartimento di Informatica, Università degli Studi di Milano, Via Celoria 18, 20133 Milano, Italy

**Keywords:** empirical mode decomposition, incremental merge segmentation, singular spectrum analysis, remote photoplethysmography, pyVHR, remote respiratory rate estimation, vital signs from video, contactless respiration monitoring

## Abstract

The respiration rate (RR) is one of the physiological signals deserving monitoring for assessing human health and emotional states. However, traditional devices, such as the respiration belt to be worn around the chest, are not always a feasible solution (e.g., telemedicine, device discomfort). Recently, novel approaches have been proposed aiming at estimating RR in a less invasive yet reliable way, requiring the acquisition and processing of contact or remote Photoplethysmography (contact reference and remote-PPG, respectively). The aim of this paper is to address the lack of systematic evaluation of proposed methods on publicly available datasets, which currently impedes a fair comparison among them. In particular, we evaluate two prominent families of PPG processing methods estimating Respiratory Induced Variations (RIVs): the first encompasses methods based on the direct extraction of morphological features concerning the RR; and the second group includes methods modeling respiratory artifacts adopting, in the most promising cases, single-channel blind source separation. Extensive experiments have been carried out on the public BP4D+ dataset, showing that the morphological estimation of RIVs is more reliable than those produced by a single-channel blind source separation method (both in contact and remote testing phases), as well as in comparison with a representative state-of-the-art Deep Learning-based approach for remote respiratory information estimation.

## 1. Introduction

Physiological signs, such as Blood Volume Pulse (BVP), Blood Pressure, Electro-Dermal Activity, blood Oxygenation levels ($SpO_{2}$) and Respiration Waveforms and Rates are of chief importance in a variety of contexts related to health monitoring and affective computing. Among them, Respiratory Rate (RR) is crucial to detect and assess respiratory dysfunctions, such as apnea [1], as well as changes in breathing patterns that may be caused by stress [2].

**Figure 1:**
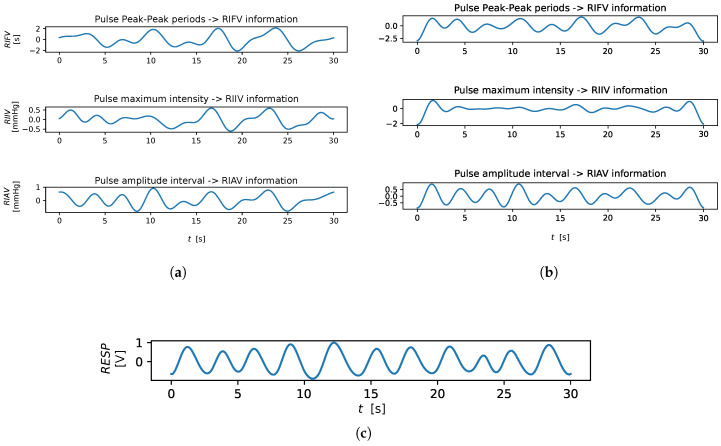
Example of RIV trends. (**a**) RIVs extracted from the contact reference. (**b**) RIVs extracted from remote-PPG. (**c**) Reference respiratory signal.

Traditionally, reliable electrocardiography sensors or respiration belts have been adopted to measure RR, but in some cases, these approaches are not feasible due to several reasons, from physical discomfort, to the need of employing dedicated equipment and expertise, up to the actual impossibility of placing the required sensors [3]. Alternative solutions rely on the adoption of photoplethysmography (PPG) sensors, such as pulse oximeters, being less invasive and augmenting the portability [4]. PPG sensors capture the reflected light skin variations due to the blood volume changes. By design, their straightforward application concerns the measurement of cardiac activity; however, due to the tight bond between cardiac and respiratory activities, signals derived from PPG waveforms may be employed to extract respiratory-related information [5]. Interestingly enough, even general-purpose smartphone cameras can function as pulse oximeters [6], thus allowing the extraction of PPG signals without the use of dedicated equipment. As a matter of fact, such a solution is much less invasive, only requiring to place a finger on the smartphone’s camera. Even more attractive are contactless techniques implementing Remote-Photoplethysmography (remote-PPG or rPPG): comfortable for the user, and suitable for remote monitoring. Remote-PPG measures blood flow changes by analysing the variations of the skin-reflected light captured through a general-purpose RGB-camera placed in front of the person [7]. While rPPG widens the scope of applications [8,9,10], its utility clearly depends on its reliability. As with contact-PPG signals, remotely estimated PPG waveforms carry respiratory-related information; there exists at least three different kinds of Respiratory Induced Variations, RIVs that can be eventually mined from (r)PPG signals: (cfr. Figure 1) [11,12,13]:

- Respiratory-Induced Intensity Variation (RIIV) refers to the modulation of the BVP signal’s amplitude caused by changes in venous return due to variations in intra-thoracic pressure during the respiratory cycle. As a result, the PPG signal experiences baseline modulation. Inspiration causes a reduction in intra-thoracic pressure, leading to a slight decrease in central venous pressure and an increase in venous return, while expiration causes the opposite effect.
- Respiratory-Induced Amplitude Variation (RIAV) is caused by a reduction in left ventricular stroke volume due to changes in intra-thoracic pressure, resulting in a decrease in cardiac output and peripheral pulse strength. During expiration, the opposite effect occurs.
- Respiratory-Induced Frequency Variation (RIFV) refers to the periodic variation of Heart Rate (HR) throughout the respiratory cycle, with an increase during inspiration and a decrease during expiration. This change in pulse rate is due to an autonomic nervous system response known as respiratory sinus arrhythmia (RSA).

The chief concern of this work is to benchmark representative approaches that allow to extract RIVs from rPPG signals and to perform a sound statistical assessment of the results on a publicly available dataset. Notably, although other works have attempted to compare such methods (see, for instance, Ref. [14]); to the best of our knowledge, this is the first attempt at analysing these methods’ performances on a publicly available dataset. The research questions that the present work aims at addressing are the following:

1. Which kind of approach is more suitable for extracting respiratory related information from rPPG?
2. What is the impact of using rPPG on the quality of estimation compared to contact-PPG?
3. How does this result compare to representative modern approaches relying on Deep Learning methods?
4. Are the eventual differences statistically significant?

Two main kinds of approaches have been proposed in the literature to achieve RR estimation via rPPG: (1) methods based on the direct extraction of morphological features attributable to breathing (RIVs) [11,12,14,15,16,17], and (2) methods aimed at isolating the motion trend due to heart rate (HR) and RR [18,19,20], implicitly related to RIVs. By and large, the reliability of these approaches has been hitherto tested on small and/or private datasets, thus preventing a fair comparison among them.

Specifically, in order to evaluate methods based on the direct RIV extraction, we addressed the well-known Incremental Merge Segmenting (IMS), presented in Ref. [21], while putting in place several solutions to fuse the produced morphological features (e.g., average, median or PCA). As to the second group, the most promising approaches adopt the single-channel blind source separation to isolate the respiration from heart rate and noise. Here, we consider the Empirical Mode Decomposition (EMD) [22] and the singular spectrum analysis (SSA), and compare their estimates based on the different channels.

In our experimental analysis, the RR estimation is evaluated on both the contact reference and remote-PPG to assess the reliability of the different approaches mentioned above. In order to make the experiments reproducible and extendable, we use the publicly available BP4D+ dataset [23] that includes the RR ground truth, the contact reference, and RGB videos suitable to estimate remote-PPG signals. This last step is accomplished by exploiting the pyVHR framework [24].

In addition, an experiment involving a deep learning-based solution has been carried out. Specifically, the aforementioned signal processing-based approaches were compared with a representative state-of-the-art Deep Learning-based approach devised to estimate cardio-respiratory information from RGB videos.

The remainder of this paper is organized as follows: in Section 2 the Photoplethysmography is introduced; in Section 3, the considered RR estimation algorithms are outlined; in Section 4, the experimental analysis is reported, and in Section 5 the results so far achieved are discussed.

## 2. Photoplethysmography and Remote-Photoplethysmography

The PPG signal conveys information concerning blood volume changes in the microvascular bed of the tissue [25]. Its waveform typically displays: (1) A pulsatile component of artery blood (AC); (2) a high-frequency component (HF), composed of a systolic phase and a diastolic phase, which provides heart rate information; and (3) a non-pulsatile component of artery blood (DC), which is a low-frequency component (LF), related to blood oxygen saturation that slowly varies with respiration. It is possible to infer the respiratory rate from a PPG signal from this slow modulation in the LF component. Contact signals in classic PPG provide intensity change measurements of the light reflected from the finger skin when exposed to a Light-Emitting Diode (LED) source.

Similarly, remote-PPG aims at characterizing the blood volume changes, but taking into account reflected light skin variations as captured by an RGB camera and focusing on a peculiar Region of Interest (ROI), such as the cheeks or the forehead. Remote-PPG can thus be conceived as an approximation of the contact reference, while offering a notable advantage: it is contactless and remotely controllable.

Generally speaking, the time signal produced by classical PPG sensors can be closely approximated by the temporal traces of the RGB signal, which are generated by averaging the skin’s light intensity at the pixel level within the ROI and concatenating them on a per-frame basis. Several methods have been proposed to derive the remote-PPG from the RGB traces [26]. Here, we adopt the CHROM method [27], since contrarily to most of the other rPPG methods, it removes specular reflections at the skin surface. The implementation of CHROM used here is part of the pyVHR framework [24,26].

The subsequent procedures involve dividing a rPPG signal or a contact reference signal x(t) of length *T* into *S* segments or windows, each of *M*-sample length, with a shift of *K* samples between adjacent segments, thus producing an overlapping of M−K samples. The *j*-th segment, with j∈[0,...,S−1], can be expressed as

(1) $$x_{j}\left(t\right)=x\left(t+jK\right),\;t=1,\cdots ,M,$$

where jK is the starting element of segment *j*.

## 3. Respiratory Rate Estimation

In this section, the operational processes and algorithmic mechanisms of the three mentioned methods, namely, Incremental Merge Segmenting (IMS), Empirical Mode Decomposition (EMD), and Singular Spectrum Analysis (SSA), are discussed in detail.

### 3.1. IMS Algorithm

IMS is an effective technique for analyzing PPG signals, extracting morphological features and detecting artifacts [6]. It operates in the time domain by segmenting the signal with sliding windows of fixed length and duration of a few tens of seconds (up to 30) (Equation (1)). From a bare technical point of view, it is a fusion of two algorithms, namely, the Iterative-End-Point-Fit (IEPF) [28] and the incremental algorithm [29]. Both of these algorithms were originally designed for computer vision, mobile robotics, and ECG signal compression applications. In a nutshell, IMS simply segments the (r)PPG signals in order to detect peaks, and subsequently obtain pulse amplitude, maximum and minimum intensity, and pulse period. These features are then employed to extract RIVs. More specifically, beats are detected at the end of a sequence of positive-gradient segments (systolic upslopes).

The IMS algorithm has been employed in Ref. [21] for the segmentation of remote-PPG signals and subsequent extraction of respiratory waveforms, as detailed below.

For each *j*-th segment, the input of the IMS algorithm are the segment values $x_{j}\left(t\right)$ and an integer parameter m<M (*m* is common to all segments). IMS uses a collection of strided points $Y_{j}=\left\{y_{k}=x_{j}\left(mk\right):k=1,\cdots ,\left\lfloor M/m\right\rfloor \right\}$ to compute morphological features. Specifically, it first computes the slope $\rho_{k}$ of each sub-segment $\mathrm{Line}[y_{k},y_{k+1}]$, and then iteratively removes from the $Y_{j}$ set all central points of the triplets $\left(y_{k},y_{k+1},y_{k+2}\right)$ that satisfy $\mathrm{sign}\left(\rho_{k}\cdot \rho_{k+1}\right)>0$, that is, slopes with the same sign. The points stored in $Y_{j}$ are finally used to obtained minima $Y_{j}^{min}$ and maxima $Y_{j}^{max}$ through peak detection.

The extraction of respiratory-induced variations (RIVs) can be easily accomplished by combining and filtering the values of $Y_{j}^{min}$ and $Y_{j}^{max}$ to effectively reduce any artifacts.

In particular, given a generic sequence of values x, the artifact reduction is obtained computing the first derivative of its cubic spline interpolation:

(2) Π(x)=Deriv(Spline(x)).

The three types of Respiratory Induced Variations can be mined from IMS-segmented PPG waveforms in the following way [30]:

- The respiratory induced intensity variation (RIIV) is conveyed by the local maximum-peak-valued time series:
  (3) $$RIIV_{j}=\Pi \left(Y_{j}^{max}\right).$$
- The respiratory induced amplitude variation (RIAV) is carried by the series generated from the difference between local maximum values and local minimum values (amplitude trend):
  (4) $$RIAV_{j}=\Pi \left({\left\{y_{i}^{max}-y_{i}^{min}\right\}}_{\forall i}\right)$$
  where $y_{i}^{max},y_{i}^{min}\in Y_{j}^{max},Y_{j}^{min}$.
- The respiratory induced frequency variation (RIFV) can be calculated by creating a tachogram composed of evenly sampled and minimum-peak-time-interspersed series, which consists of the time intervals between consecutive local minima:
  (5) $$RIFV_{j}=\Pi \left({\left\{\arg\left(y_{i}^{min}\right)-\arg\left(y_{i+1}^{min}\right)\right\}}_{\forall i}\right)$$
  where $y_{i}^{min}\in Y_{j}^{min}$ and arg$\left(y_{i}^{min}\right)$ are the time instants associated to the *i*-th value in $Y_{j}^{min}$.

**Figure 2:**
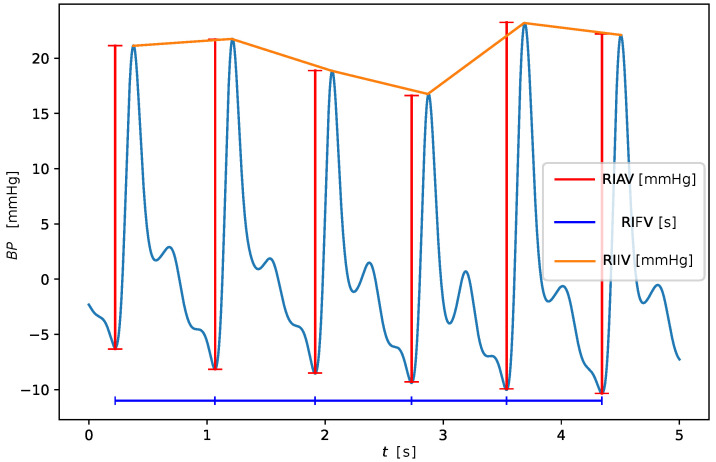
Example of derivation of the respiratory induced variations capturing the amplitude (RIAV), the frequency (RIFV) and the intensity (RIIV) from the pulse subdivision of the PPG waveform.

An example of extracted RIVs, from both contact and remote PPG, is shown in Figure 1, while Figure 2 shows at a glance the three RIVs on a sample cardiac signal from the BP4D+ dataset.

The $RIFV_{j}$, $RIIV_{j}$, and $RIAV_{j}$ extracted from (r)PPG signals can be used individually to generate remote RR estimations or combined to obtain more reliable estimates [14,15,31]. To this end, besides the average and the median over the three RIVs, PCA is computed considering the RIVs as an ensemble of realizations of the respiratory trend. RIVs are therefore projected onto their principal component, and only the first principal component is taken into account for the RR estimation.

This process produces six estimates: $RR_{RIFV}$, $RR_{RIIV}$, $RR_{RIAV}$, $RR_{avg}$, $RR_{median}$, and $RR_{PCA}$.

### 3.2. EMD: Empirical Mode Decomposition

Empirical mode decomposition (EMD) is a powerful analytical tool used to effectively describe non-linear and non-stationary time series. This approach involves projecting the time series onto a space basis composed of intrinsic mode functions (IMFs) [22]. Unlike Fourier Transforms and wavelet decomposition, EMD works entirely within the time domain to decompose the data into its constituent IMFs, and it does not require any prior assumptions about the signal’s frequency or time-frequency characteristics. Instead, it adaptively decomposes the signal based on its local frequency content using a a time-domain algorithm called “sifting”, capturing the underlying dynamics of the system. In a nutshell, EMD isolates a small number of temporally adaptive basis functions (the IMFs) and directly derives the frequency and amplitude dynamics from them [32]. The IMFs can be thought of as locally changing counterparts to common frequencies. This characteristics of IMFs make them a valuable tool for analyzing non-stationary time series with rapidly varying frequencies, allowing for the accurate identification and extraction of frequency and amplitude dynamics. More precisely, an IMF is defined as a function that satisfies well-defined conditions related to its Hilbert transform, and summarized in the following two properties:

1. The number of extrema (i.e., the maximum and minimum amplitudes of the signal) and the number of zero-crossings must be equal, or differ by one at most. This property ensures that the function is oscillatory in nature, with a well-defined and localized frequency structure that can be accurately extracted using techniques such as the Hilbert transform.
2. The function must be symmetric with respect to a local zero mean. The need for a local time scale to calculate the mean makes it challenging to define a function as symmetric for non-stationary processes. To solve this problem, the local mean envelope concept is introduced, which is determined by the function’s local maximum and minimum values. By enforcing local symmetry around this envelope, the IMF can be accurately characterized and used to extract information about the underlying dynamics of the system.

In formal terms, given an arbitrary non-stationary PPG signal x(t), EMD adaptively decomposes its segments $x_{j}\left(t\right)$ into a number *L* of IMFs $h_{j}^{k}\left(t\right)$, with 1≤k≤L, that is,

(6) $$x_{j}\left(t\right)=\sum_{k=1}^{L}h_{j}^{k}\left(t\right)+r_{j}\left(t\right),$$

where $r_{j}\left(t\right)$ represents the residual non-stationary trend. The iterative sifting process to derive a generic *k*-th IMF function is summarized in the following iterative steps. As initialization, we set the data to be processed as $v\left(t\right)=x_{j}\left(t\right)$, k=1, i=0.

1. Extract the local maxima and minima of v(t).
2. Form the upper and lower envelope $e^{u}\left(t\right)$ and $e^{l}\left(t\right)$ by cubic spline interpolation of the extrema, and compute the mean $m\left(t\right)=(e^{l}\left(t\right)+e^{u}\left(t\right))/2$.
3. Let $d_{i}\left(t\right)=v\left(t\right)-m\left(t\right)$.
4. When i>1, evaluate whether $d_{i}\left(t\right)$ is a zero-mean function. This is obtained in terms of the standard deviation of two subsequent iteration results:
  (7) $$\sum_{t=1}^{M}\frac{{\left(d_{i}\left(t\right)-d_{i-1}\left(t\right)\right)}^{2}}{d_{i-1}{\left(t\right)}^{2}}\leq 0.1\;.$$
  If the standard deviation exceeds a fixed threshold (set to 0.1, according to [32]), set $v\left(t\right)=d_{i}\left(t\right)$ as the new data, increment *i*, and repeat steps 1–4 until ending up with the *k*-th IMF, that is, $h_{j}^{k}\left(t\right)=d_{i}\left(t\right)$.

Once the *k*-th IMF is obtained, the remaining IMFs were computed by applying the sifting process to the residual signal defined as $r\left(t\right)=v\left(t\right)-h_{j}^{k}\left(t\right)$, repeating the steps 1–4 to compute the next IMF, until the *n*-th residual is a monotonic function or a function with less than two local maxima or minima.

**Figure 3:**
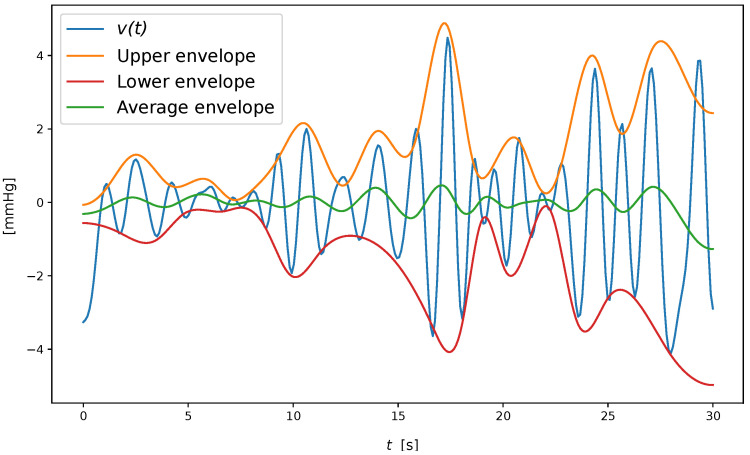
Example of envelopes involved in an iteration of $h_{j}^{k}\left(t\right)$ computation.

**Figure 4:**
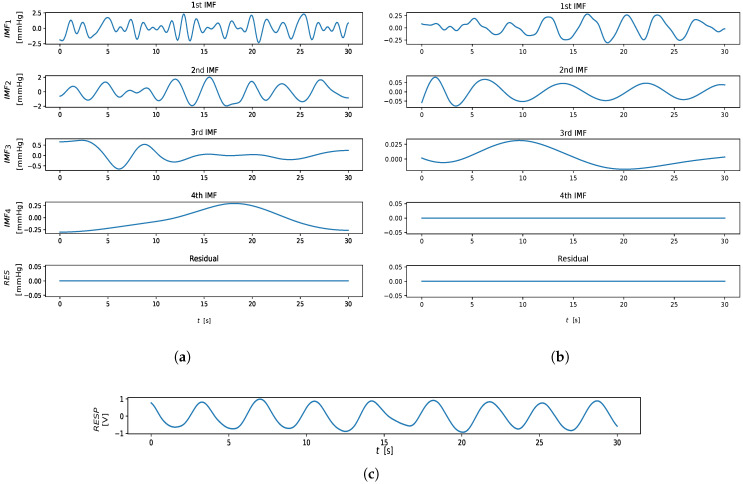
Extracted from IMF trends. (**a**) IMFs extracted from the contact reference. (**b**) IMFs extracted from rPPG. (**c**) Thoracic-impedance respiratory reference.

An example of $h_{j}^{k}\left(t\right)$ computation is shown in Figure 3. From an implementation perspective, we adopted the solution provided by Ref. [32], limiting the number of IMFs extracted to four components, as suggested in Ref. [21]. The output of the EMD algorithm is shown in Figure 4.

For the RR extraction, only the first three IMFs, among the IMFs extracted through the EMD algorithm, were taken into account.

The $RR_{IMFi}$ estimate is obtained for each IMF mode by selecting the frequency in the respiratory spectral domain with the highest power. The highest peaked frequency among the estimates $RR_{IMF1}$, $RR_{IMF2}$, $RR_{IMF3}$, $RR_{IMF4}$ is taken as the $RR_{EMD}$ estimate.

### 3.3. SSA: Singular Spectrum Analysis

Singular spectrum analysis [33] is a decorrelation technique that projects a single mixture of zero-mean sources (time series) onto an orthonormal space basis. It involves decomposing the time series (PPG in our case) into a set of empirical orthogonal functions (EOFs) by constructing a trajectory matrix from the original data and applying the Singular Value Decomposition (SVD) to the matrix. The result is a set of principal components (PCs) that capture the most dominant patterns in the data. These PCs are then used to reconstruct the original time series in a way that separates the signal from the noise.

To perform the embedding, we mapped a PPG segment $x_{j}\left(t\right)$ of length *M* into a sequence $X_{j}^{i}$ of K=M−L+1 lagged vectors of size *L*, with 1<L≤M:

(8) $$X_{j}^{i}={\left(x_{j}\left(i\right),\cdots ,x_{j}\left(i+L-1\right)\right)}^{T},\;1\leq i\leq K.$$

The *L*-trajectory matrix *X* associated to $x_{j}\left(t\right)$ is

(9) $$X={\left(x_{i,j}\right)}_{i,j=1}^{L,K}\left[\begin{array}{cccc} x_{j}\left(1\right) & x_{j}\left(2\right) & \ldots & x_{j}\left(K\right)\\ x_{j}\left(2\right) & x_{j}\left(3\right) & \ldots & x_{j}\left(K+1\right)\\ . & . & . & .\\ . & . & \;\;\;\;. & .\\ x_{j}\left(L\right) & x_{j}\left(L+1\right) & \ldots & x_{j}\left(M\right), \end{array}\right]$$

and the lagged vectors $X_{j}^{i}$ represent the columns of *X*. Each row and column of *X* consists of subseries of the original segment (or series) $x_{j}\left(t\right)$. It holds that the trajectory matrix *X* is a Hankel matrix, where the elements along any diagonal where i+j is constant have the same value.

The basic steps of SSA are as follows.

**Figure 5:**
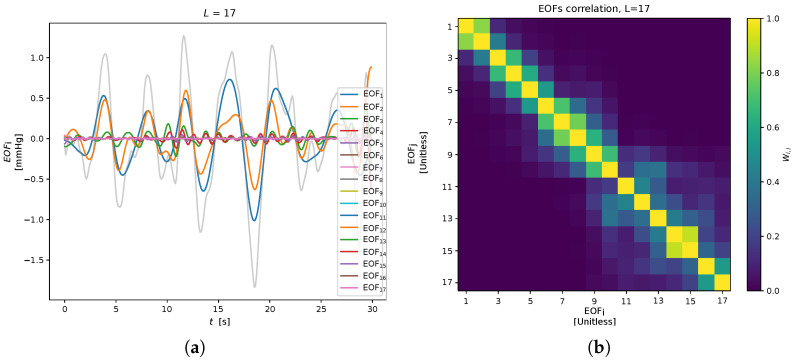
SSA produces *L* empirical orthogonal functions, which are statistically correlated to each other in accordance with the weighted correlation matrix. (**a**) EOFs (L=17). (**b**) Weighted correlation matrix.

**Figure 6:**
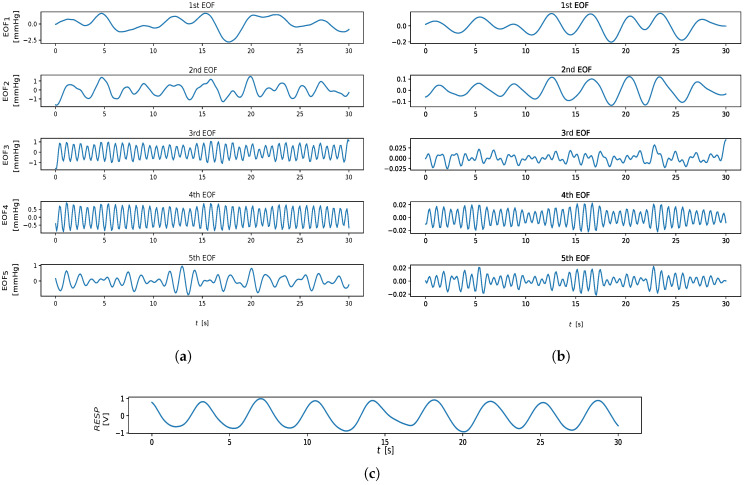
Extracted EOF trends. (**a**) EOFs extracted from rPPG. (**b**) Thoracic-impedance respiratory reference. (**c**) Thoracic-impedance respiratory reference.

1. Embedding. The realization $x_{j}\left(t\right)$ is embedded into a trajectory matrix *X* using the sliding window approach described above.
2. SVD decomposition. To obtain the principal components, apply SVD to the trajectory matrix *X*, as $X=U\;\Sigma \;V^{T}$, where *U* is the matrix of the eigenvectors (left singular vectors), Σ is the diagonal matrix of the singular values $\left(\lambda_{1},\cdots ,\lambda_{L}\right)$ and *V* is the matrix of the right singular vectors of *X*. In this notation, the trajectory matrix *X* can be written as
  (10) $$X=X_{1}+\cdots +X_{d},$$
  where $X_{i}=\sqrt{\lambda_{i}}U_{i}V_{i}^{T}$ is the elementary matrix, and d=min{K,L} is the rank of *X* (matrices $X_{i}$ have rank 1). The sequence of elements of the *i*-th eigenvector $U_{i}$ is defined as the *i*-th Empirical Orthogonal Function (EOF) (see Figure 5).
3. Grouping. The principal components are grouped into sets, aiming at representing the different patterns present in the data (e.g., noise, periodicity, trend). This corresponds to partitions in the set of indices {1,⋯,d} into *p* disjoint subsets $S_{1},\cdots ,S_{p}$.
4. Reconstruction. Given a subset of indexes $S_{i}=\left\{i_{1},...,i_{l}\right\}$, an approximation of *X* is reconstructed as a sum of the corresponding elementary matrix only: ${\hat{X}}_{i}=X_{i_{1}}+\cdots +X_{i_{l}}$. Then, applying the Hankelization of ${\hat{X}}_{i}$, the time series $z_{i}=\left(z_{1},\cdots ,z_{M}\right)$ is obtained. For the sake of brevity, Hankelization and averaging are not reported here; see Ref. [34] for details.
  The SSA output, for the remote and contact analysis, is reported in the Figure 6. Here, no PC grouping procedure was employed (i.e., p=d) taking into account the EOFs individually. In particular, only the first three EOFs were considered for the RR extraction: $RR_{EOFi}$ is estimated as the highest peak in the respiratory power spectral density range of $z_{i}$. Then, the highest estimate among the $RR_{EOF1}$, $RR_{EOF2}$, $RR_{EOF3}$ was chosen as the $RR_{SSA}$ value.

## 4. Experimental Analysis and Results

### 4.1. Dataset

The study was carried out employing the BP4D+ dataset [23], since it is one of the few available datasets that provides face video frames, contact references, and a real respiratory ground truth for each subject. The BP4D+ dataset, an extension of the BP4D database, is a Multimodal Spontaneous Emotion Corpus (MMSE), which collects 3D, 2D, thermal, and physiological data sequences (e.g., heart rate, blood pressure, skin conductance (EDA), and respiration rate), and meta-data (facial features and FACS codes). The dataset collects data of 140 subjects, 58 males and 82 females, with ages ranging from 18 to 66 years old. Subjects of various ethnicities participated in the data acquisition process (including East-Asian and Middle-East-Asian, Hispanic/Latino and Native American). For each subject, 10 tasks (eliciting different emotional states) were included in the database. Frames were acquired at 25 fps, and physiological signals were sampled at 1000 Hz. For the aim of this study, only the thoracic-impedance respiratory reference, the contact reference, and the subject’s face video frames were taken into account, considering all the subjects and all the tasks.

As stated in Ref. [31], some signals in the BP4D+ respiratory ground truth are strongly affected by artifacts and disturbance, therefore the thoracic-impedance respiratory reference signals were pre-processed to check their reliability as a respiratory target. For each task and subject, the respiratory reference was filtered with a second-order Butterworth bandpass filter, with cutoff frequencies of 0.1 Hz and 0.5 Hz, and normalized between the [−1,1] amplitude range. Then peak and troughs detection was performed, so that the standard deviation $\sigma_{1}$ of the peak-to-peak time intervals and the standard deviation $\sigma_{2}$ of the heights of the troughs (minima) could be computed. A signal was considered "corrupted" if $\sigma_{1}>1$ or $\sigma_{2}>0.2$ (leading to the rejection of the related data). Additionally, each signal with a duration shorter than 30 s was discarded. This results in 209 accepted signals.

### 4.2. Analysis

**Figure 7:**
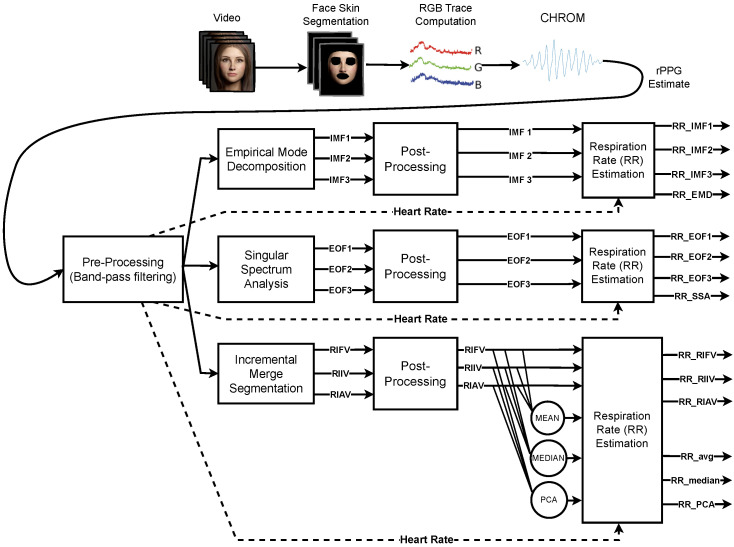
Computation pipeline. First line: steps to obtain the rPPG via pyVHR framework. Following lines: EMD/SSA/IMS processes to derive the RR estimations either based on the individual features (IMF, EOF, and RIV) or combining them opportunely. Pre-processing filtering and artifact reduction are setups for all methods.

The single-channel blind source separation methods (EMD and SSA) and the RIV extraction technique via (r)PPG signal segmentation (IMS) are adopted during the RR estimation phase, processing the remote PPGs and contact references of each BP4D+ video. The general procedure for computing RR is depicted at a glance in Figure 7, and can be briefly recapped as follows:

1. Pre-processing: The rPPG signal associated with the *i*-th video is filtered using a fourth-order Butterworth band-pass filter with cut-off frequencies of [0.18 Hz, 1.0 Hz] for each temporal window indexed by *j*.
2. Methods: The filtered signal is processed by one of the analysed approaches (IMS, EMD, SSA) in order to extract respiratory related information.
3. Post-processing: Each approach yields a number of estimates that are subsequently post-processed with an artifact reduction technique. It basically consists of a cubic-spline interpolation of the original estimate followed by the computation of the fist derivative of the obtained signal (cfr. Equation (2)).
4. RR estimation: RR is obtained by choosing the most prominent peak in the Power Spectral Density (PSD) estimated with the periodogram of the post-processed signal. The frequency range within which RR is picked is adaptively set, based on the heart rate extracted from the same signal.

The obtained RR-estimates are then compared to the corresponding ground truth references via commonly used error metrics. Contact references are processed in the same way, except for the *post-processing* phase, which involves only the computation of the fist derivative of the signal.

#### 4.2.1. Error Metrics

For each estimator, the obtained RR estimate is compared to the RR extracted from the thoracic-impedance respiratory reference using the following metrics:

- Mean Absolute Error (MAE) The Mean Absolute Error measures the average absolute difference between the estimated $\hat{h}$ and reference RR *h*. It is computed as:
  $$\mathrm{MAE}=\frac{1}{K}\sum_{k}\left|{\hat{h}}^{k}-h^{k}\right|.$$
  Smaller MAE values suggest better predictions. The MAE is a fairly interpretable measure, as it provides the average distance in terms of breaths per minute of the predictions with regard to the ground truth.
- Root Mean Squared Error (RMSE). The Root-Mean-Squared Error measures the difference between quantities in terms of the square root of the average of squared differences, that is,
  $$\mathrm{RMSE}=\frac{1}{K}\sqrt{\sum_{k}{\left({\hat{h}}^{k}-h^{k}\right)}^{2}}.$$
  RMSE represents the sample standard deviation of the absolute difference between the reference and measurement, that is, a smaller RMSE suggests more accurate extraction. In contrast to the MAE, few large differences increase the RMSE to a greater degree due to the squaring of the differences.

Results for all the aforementioned estimators applied to both contact and remotely estimated signals are reported in Table 1.

#### 4.2.2. Bland–Altman Analysis

In order to assess the level of agreement between the employed rPPG-based RR estimation methods and the reference, Bland–Altman analysis [35] has been employed. It allows to quantify the difference between measurements using a graphical method. A scatter-plot (Bland–Altman plot) is produced in which the X-axis represents the average of the two measurements, and the Y-axis represents their difference.

For every pair ($RR_{estimator},RR_{target}$), the mean value (i.e., what is likely to be interpreted as a RR trade-off between expectation and the true value) versus the corresponding error (i.e., how reliable the compromise is for the RR measurement) are considered:

(11) $$Error_{RR}=RR_{estimator}-RR_{target}$$

The sign of this quantity allows to unveil the presence of eventual systematic biases in the estimation. Specifically, negative errors indicate that the RR is, on average, underestimated, while positive errors suggest that the RR is typically overestimated.

**Figure 8:**
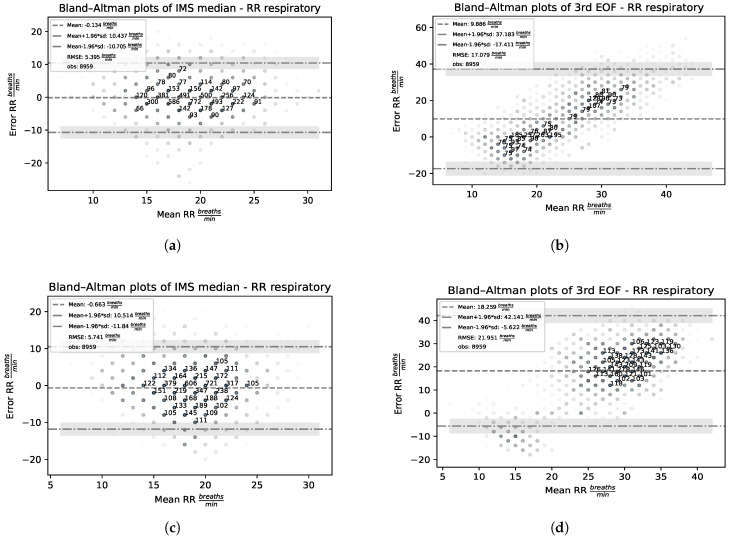
Bland–Altman plots for the $RR_{median}$ and $RR_{EOF3}$ estimators on contact and remote−PPG signals. (**a**) Contact $RR_{median}$. (**b**) Contact $RR_{EOF3}$. (**c**) Remote $RR_{median}$. (**d**) Remote $RR_{EOF3}$.

Figure 8 reports Bland–Altman plots for the $RR_{median}$ and $RR_{EOF3}$ estimators acting on contact and remote-PPG signals.

As can be noted, the $RR_{median}$ estimator (Figure 8a,c) is unbiased (${\mathrm{Error}}_{RR}∼0$) and does not exhibit any linear dependency between the average of the two measurements and their difference for both the contact and remote signals. On the contrary, an inspection of the $RR_{EOF3}$ Bland–Altman plot (Figure 8b,d) depicts a biased estimator (estimates are, on average, heavily overestimated) which presents a marked linear dependence between the average of the two measurements and their difference. Consequently, for low-frequency reference RR, $RR_{EOF3}$ underestimates RR, while overestimating high-frequency reference RR.

#### 4.2.3. Significance Testing

Finally, statistical analyses have been carried out to evaluate the significance of differences in the performance of the 14 estimators (populations) on 209 samples (paired videos). Following Ref. [36], the populations were checked with the Shapiro–Wilk test for normality, and with Levene’s test for homogeneity. Due to the rejection of the null hypothesis of normality for at least one population, the non-parametric Friedman’s test and the associated Nemenyi test were employed as the omnibus test and for post hoc analysis, respectively.

**Figure 9:**
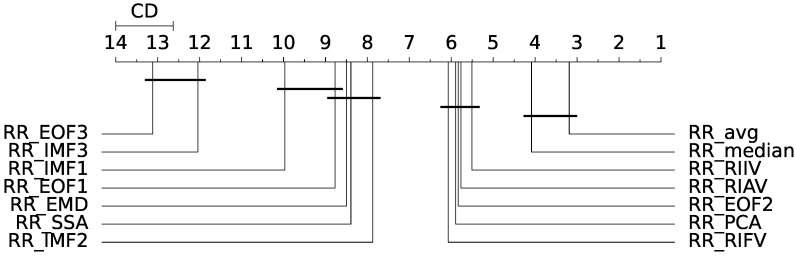
Critical difference (CD) diagram for remote measurement (with RMSE as evaluation metric).

**Figure 10:**
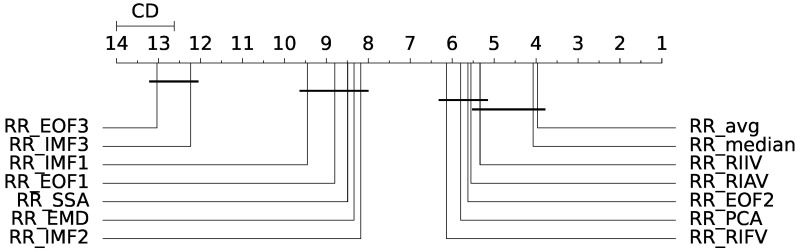
Critical difference (CD) diagram for remote measurement (with MAE as evaluation metric).

Friedman’s test rejected the null hypothesis of equality of the medians of the population distributions (p<0.05); hence, a statistically significant difference exists between the analysed estimators. The Nemenyi post hoc test was thus employed for the assessment of the differences between each population. The output of the post hoc Nemenyi test can be visualized through the Critical Difference (CD) diagram [36]; CD diagrams show the average rank of each estimator (higher ranks meaning lower average errors); models whose difference in ranks do not exceed the CD${}_{\alpha }$ (α=0.05) are joined by thick lines and cannot be considered significantly different. Results for the MAE and RMSE metrics obtained from the remote-PPG signal are depicted at a glance in Figure 9 and Figure 10.

Table 2 and Table 3 report the results of the above procedure for the RMSE and MAE metrics, respectively. Estimators are ranked with regard to the remote-RR estimation performances according to the metric at hand. Moreover, the magnitude of the difference between the estimators is reported in terms of Akinshin’s gamma effect size.

#### 4.2.4. Comparison with a Deep Learning-Based Approach

The approaches evaluated in this work so far pledge to extract respiratory-related information by exploiting the well-known intertwining between the cardiac and respiratory system; on such basis, the signal processing-based methods surveyed here allow to extract the physiological signals of interest with varying levels of accuracy. It is interesting to confront such approaches relying on domain knowledge with modern alternatives that allow to automatically learn these relationships from data; this is usually accomplished by employing end-to-end deep neural network (DNN) models. DNNs have gained significant attention in many disciplines, including computer vision, in virtue of their superior performance exhibited for a variety of tasks when compared to traditional approaches that require manual feature design.

Notably, techniques for recovering physiological signals via DNN-based methods have also emerged. The latter have become increasingly popular in the related literature, as evidenced by recent reviews, for example, Refs. [37,38]. Despite the reported remarkable performances, few DNN solutions have been made publicly available in terms of both code and learned model weights. This lack of availability raises concerns about the reproducibility of results and the ability to properly assess these methods.

Deep remote RR estimation makes no exception; in the last few years, a variety of approaches have been proposed for predicting respiratory rates or waveforms directly from RGB videos [39,40,41,42]. Unfortunately, to the best of our knowledge, only a handful of them have been made available to the scientific community. One of them is represented by MTTS-CAN, a neural architecture proposed in Ref. [40], which employs a tensor-shift module and 2D-convolutional operations to efficiently perform spatial temporal modeling, thus enabling real-time cardiovascular and respiratory measurements. MTTS-CAN is end-to-end trained to predict both Blood Volume Pulse (BVP) signals and Respiratory Waveforms (RWs) from videos displaying human faces. Model optimization is shaped as a multi-task learning problem. At test time, BVPs as RWs can be easily obtained by feeding the network with a given video sequence; no pre-processing steps are required, except for performing trivial image normalization procedures.

It is worth noting that MTTS-CAN is designed to work in an end-to-end manner and does not estimate respiratory information from rPPG signals, as intended. Yet, the multi-task temporal shift module employed to extract both cardiac and respiratory information is eventually capable of leveraging both sources in order to deliver more robust estimates. Consequently, it lends itself well to be compared with the approaches presented here.

**Figure 11:**
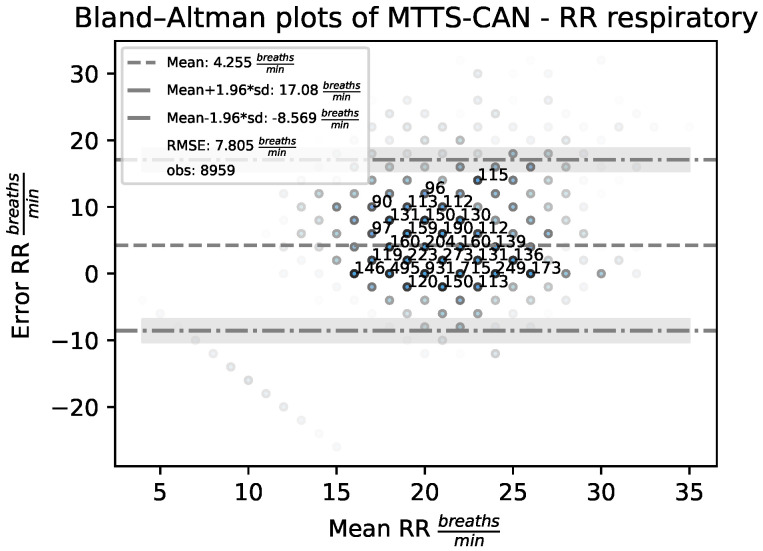
Bland–Altman plots for the $RR_{MTTS-CAN}$ estimator on remote signals.

Given a RW estimated by MTTS-CAN, RR can be eventually obtained via spectral analysis (cfr. point 4 in Section 4.2). The Bland–Altman plot depicting the results obtained by MTTS-CAN on the BP4D+ dataset is displayed in Figure 11.

As can be observed, MTTS-CAN tends, on average, to slightly overestimate the respiration rates on BP4D+; on the contrary, no marked linear dependencies between the average of the two measurements and their difference are noticeable. The standard deviation of the errors is comparable with the best-performing signal processing-based approaches.

**Figure 12:**
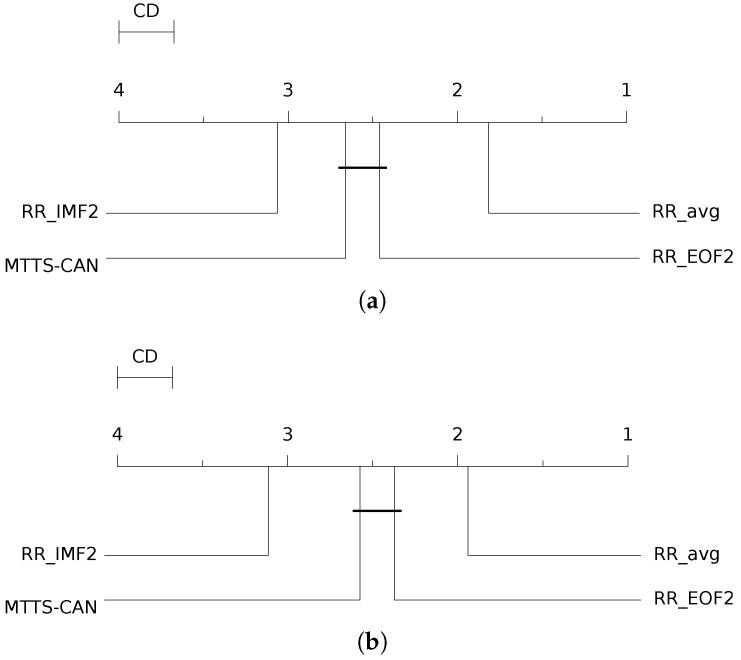
CD diagram displaying the results of the Nemenyi post hoc test on the four populations ($RR_{avg}$, $RR_{EOF2}$ and $RR_{IMF2}$ and MTTS-CAN) of RMSE and MAE values on the BP4D+ dataset. (**a**) RMSE. (**b**) MAE.

In addition, to compare knowledge-based signal processing techniques with DNN-based approaches, we follow the same experimental and significance testing procedure, as described earlier in Section 4.2.3. Specifically, we compare the RR estimates produced by MTTS-CAN and benchmark its performance against the three best estimators from each method examined in this study ($RR_{avg}$, $RR_{EOF2}$, and $RR_{IMF2}$). We present the results in Table 4 and include the corresponding CD diagrams for both the MAE and RMSE metrics in Figure 12.

Surprisingly, despite a fairly acceptable ranking with regard to the signal processing-based approaches, the RR estimates delivered by MTTS-CAN appear to be significantly less accurate than those obtained by the $RR_{avg}$ estimator; the latter proves to be the most reliable approach among those benchmarked in the present work. MTTS-CAN results are not significantly different from those yielded by the $RR_{EOF2}$ estimator, while the EMD-based estimator delivers the worst result.

## 5. Discussion and Conclusions

This paper has conducted a statistical evaluation of prominent methods for remotely estimating respiratory rates through rPPG signals. Specifically, we compared the performances of the IMS algorithm (a well-known method extracting morphological PPG features related to respiratory information) and two single-channel BSS approaches. Experiments have been carried out on a publicly available dataset, promoting greater comparability and reproducibility of the findings.

The results can be best summarized by inspecting Figure 9; the $RR_{avg}$ and $RR_{median}$ estimators are the best-performing. Notably, the difference with the other estimators appears to be statistically significant. No significant differences were found between $RR_{avg}$ and $RR_{median}$; yet, these significantly outperform $RR_{PCA}$ (albeit with a small magnitude effect size). Overall, according to the RMSE metric, the IMS-derived estimators delivered the most accurate results.

The remote SSA-derived $RR_{EOF2}$ estimator performs fairly well in comparison to the IMS-derived ones; this is due to the fact that the second EOF extracted by SSA is presumably devoted to the extraction of the RIIV (cfr. Figure 6). For the same reason, $RR_{IMF2}$ performed quite well for both contact and remote methods, while on average, the other EMD-related estimators performed worse with regard to the other estimation methods. Interestingly enough, the third EOF and the first IMF both exhibited bad results, as probably often associated with the cardiac information rather that the respiratory one (crf. Figure 1 and Figure 6). $RR_{IMF3}$ strongly underestimates the RR, meaning that it is heavily affected by low-frequency artifacts. Similar conclusions can be drawn by inspecting the MAE-related CD diagram (Figure 10).

Furthermore, we have conducted a comparison between the three top-performing signal processing-based methods for extracting respiratory information from rPPG waveforms and a newly proposed, state-of-the-art deep learning-based method apt at jointly estimating cardio-respiratory data from RGB videos. The findings indicate that the method based on extracting and merging Respiratory-Induced Variations (RIVs) from morphological features achieves the highest accuracy in comparison to the other signal processing or Deep Learning-based methods adopted here, as measured by both RMSE and MAE metrics.

Based on the assessment conducted in this paper, it can be concluded that the estimation of RR using morphological features of the PPG signals is the most dependable method. Moreover, it can be observed that the three RIVs basically convey the same information, which is mostly related to respiration, while being differently dependent on the subject’s individual RR, gender or age [31], as well on the subject’s health. In order to attenuate this dependency, and to make the estimation less susceptible to interference (largely when the subject’s motion is detected in the measurement), the application of fusion methods (mean, median or PCA) should be preferred.

For what concerns the single channel blind source separation techniques (SSA, EMD) the main problem is that, depending on the spectral characteristics of the artifacts in the recorded phenomena, nothing guarantees that the respiratory oscillation will be always displayed by the same IMF or the same EOF. Results show that the second IMF or the second EOF modes are the most suitable sources of respiration-related oscillations; yet, this does not provide a guarantee—results might depend on the number of low-frequency artifact sources that affect the signal and on the specific frequency subrange in which the true RR falls (i.e., the current physiological conditions of the monitored subject). This problem has been identified in the EMD literature with the terms “mode mixing”, when a single source contains different oscillatory modes which actually are separated sources, and “mode splitting”, when an actual single source is displayed in different extracted sources [43].

Although some improved techniques (such as the complementary ensemble EMD, the complete EEMD, the partly EEMD, the noise-assisted multivariate EMD, NA-MEMD, and the fast multivariate EMD, FMEMD) have been proposed to deal with mode mixing and mode splitting, the problem is still open [44]. In particular, for what concerns respiration, mode splitting is mainly caused by the fact that respiration is a spontaneously modulated phenomena that spreads differently in the low-frequency spectrum range, according to physical, cognitive and emotional demands; thus, it is often “naturally” split in time-subsequent different modes. Tracking those splits could be trickier than tracing respiration in another way.

Lastly, upon comparing the results obtained from the use of contact and remote PPGs, we noticed only a minor variation. To quantify this remark, it is worth noting that the estimates achieved by the most successful method, IMS (first six rows of Table 1) adopting either contact or remote PPGs, have an average difference of 0.47 breaths/min and 0.53 breaths/min in RMSE and MAE, respectively. Notably, the adoption of remote PPG only involves a negligible worsening of performance.

To summarize, the experiments reported here by and large show that, on the adopted dataset, the approach based on the extraction of respiration-related morphological features from (r)PPG signals should be preferred over single-channel blind source separation techniques (either SSA or EMD). More specifically, the estimation and fusion of the three Respiratory Induced Variations (RIVs) from (r)PPG signals (RIIV, RIFV, RIAV) allow for the achievement of the best results in terms of both RMSE and MAE. Empirical Mode Decomposition (EMD) performed worse on our benchmark, despite being one of the most widely employed single-channel source separation techniques for the extraction of respiratory information from PPG signals. Conversely, Singular Spectrum Analysis allowed us to separate respiratory information with higher levels of accuracy. Interestingly enough, SSA estimates performed similarly to a state-of-the-art pre-trained Deep Learning-based method. Notably, the difference in performances between the latter and the best-performing signal processing-based approach resulted to be significant with a medium effect size. Ultimately, the rPPG has been shown to result in only a slight decrease in performance when compared to contact PPG, thus enabling its dependable and extensive applicability.

## Figures and Tables

**Table 1:** Error metrics: contact PPG—respiratory reference and remote PPG—respiratory reference (evaluated over the 209 BP4D+ accepted signals).

	Contact		Remote	
	RMSE *	MAE *	RMSE *	MAE *
$RR_{\mathrm{avg}}$	5.064	3.615	5.413	4.019
$RR_{\mathrm{median}}$	5.395	3.709	5.741	4.095
$RR_{\mathrm{PCA}}$	5.593	3.796	6.307	4.656
$RR_{\mathrm{RIFV}}$	6.285	4.559	6.453	4.821
$RR_{\mathrm{RIIV}}$	5.528	3.906	6.538	4.721
$RR_{\mathrm{RIAV}}$	6.052	4.130	6.287	4.586
$RR_{\mathrm{EMD}}$	14.205	10.373	9.599	7.754
$RR_{\mathrm{IMF}1}$	19.773	6.775	13.568	10.084
$RR_{\mathrm{IMF}2}$	6.219	4.638	9.050	7.625
$RR_{\mathrm{IMF}3}$	12.235	11.292	13.626	12.799
$RR_{\mathrm{SSA}}$	10.441	7.243	9.406	7.894
$RR_{\mathrm{EOF}1}$	19.568	14.672	9.804	8.086
$RR_{\mathrm{EOF}2}$	19.350	14.301	7.994	5.456
$RR_{\mathrm{EOF}3}$	17.079	13.170	21.951	20.059

* Measured in $\frac{\mathrm{breaths}}{\min}$.

**Table 2:** Significance statistical test, using RMSE as the comparison metric, on 14 estimators (populations) and 209 samples (paired videos).

	RMSE									
	Contact					Remote				
	R	MED *	CI	γ	Magnitude	R	MED *	CI	γ	Magnitude
$RR_{\mathrm{avg}}$	1°	3.509	[2.744, 4.696]	0.000	negligible	1°	4.108	[3.350, 5.040]	0.000	negligible
$RR_{\mathrm{median}}$	2°	3.700	[2.828, 5.067]	−0.079	negligible	2°	4.551	[3.818, 5.479]	−0.204	small
$RR_{\mathrm{PCA}}$	3°	4.237	[3.169, 5.584]	−0.269	small	6°	5.218	[4.637, 6.170]	−0.493	small
$RR_{\mathrm{RIFV}}$	7°	4.980	[3.869, 6.016]	−0.531	medium	7°	5.333	[4.627, 6.164]	−0.571	medium
$RR_{\mathrm{RIIV}}$	4°	4.077	[3.266, 5.408]	−0.233	small	3°	5.198	[4.361, 6.435]	−0.472	small
$RR_{\mathrm{RIAV}}$	5°	4.844	[3.464, 6.000]	−0.473	small	4°	5.280	[4.522, 6.267]	−0.567	medium
$RR_{\mathrm{EMD}}$	10°	12.318	[9.229, 15.486]	−1.727	large	10°	7.950	[6.941, 9.560]	−1.367	large
$RR_{\mathrm{IMF}1}$	14°	19.575	[16.125, 21.906]	−2.765	large	12°	11.719	[9.613, 14.338]	−1.539	large
$RR_{\mathrm{IMF}2}$	6°	5.103	[4.053, 6.289]	−0.647	medium	8°	7.578	[6.154, 9.369]	−1.128	large
$RR_{\mathrm{IMF}3}$	9°	11.399	[10.259, 12.435]	−3.068	large	13°	12.579	[11.624, 14.000]	−2.982	large
$RR_{\mathrm{SSA}}$	8°	7.754	[6.331, 9.761]	−1.163	large	9°	8.300	[7.000, 9.401]	−1.489	large
$RR_{\mathrm{EOF}1}$	11°	18.000	[11.168, 22.527]	−1.509	large	11°	8.422	[7.135, 9.677]	−1.474	large
$RR_{\mathrm{EOF}2}$	12°	17.300	[11.937, 23.992]	−1.407	large	5°	6.074	[4.642, 7.134]	−0.751	medium
$RR_{\mathrm{EOF}3}$	13°	13.968	[11.478, 18.615]	−1.658	large	14°	21.126	[18.924, 23.749]	−3.737	large

* Measured in $\frac{\mathrm{breaths}}{\min}$.

**Table 3:** Significance statistical test, using MAE as the comparison metric, on 14 estimators (populations) and 209 samples (paired videos).

	MAE									
	Contact					Remote				
	R	MED *	CI	γ	Magnitude	R	MED *	CI	γ	Magnitude
$RR_{\mathrm{avg}}$	2°	2.904	[2.207, 3.837]	0.039	negligible	1°	3.422	[2.729, 4.178]	0.000	negligible
$RR_{\mathrm{median}}$	1°	2.978	[2.116, 3.973]	0.000	negligible	2°	3.556	[2.826, 4.329]	−0.064	negligible
$RR_{\mathrm{PCA}}$	3°	3.244	[2.286, 4.273]	−0.117	negligible	6°	4.065	[3.465, 5.188]	−0.298	small
$RR_{\mathrm{RIFV}}$	7°	4.000	[3.022, 5.087]	−0.442	small	7°	4.258	[3.644, 5.125]	−0.392	small
$RR_{\mathrm{RIIV}}$	4°	3.244	[2.500, 4.216]	−0.124	negligible	3°	4.169	[3.429, 5.176]	−0.330	small
$RR_{\mathrm{RIAV}}$	5°	3.625	[2.575, 4.797]	−0.270	small	4°	4.141	[3.359, 4.978]	−0.352	small
$RR_{\mathrm{EMD}}$	8°	9.778	[7.333, 12.021]	−1.464	large	9°	6.812	[5.771, 8.400]	−1.115	large
$RR_{\mathrm{IMF}1}$	14°	17.903	[14.400, 20.857]	−2.448	large	12°	9.264	[7.146, 12.000]	−1.342	large
$RR_{\mathrm{IMF}2}$	6°	4.178	[3.200, 5.135]	−0.515	medium	8°	6.933	[5.312, 8.649]	−1.117	large
$RR_{\mathrm{IMF}3}$	10°	10.960	[9.867, 12.133]	−3.102	large	13°	12.323	[11.333, 13.773]	−3.085	large
$RR_{\mathrm{SSA}}$	8°	6.092	[4.578, 8.090]	−0.919	large	10°	7.714	[6.065, 8.711]	−1.499	large
$RR_{\mathrm{EOF}1}$	9°	13.111	[8.350, 19.822]	−1.132	large	11°	7.689	[6.133, 9.131]	−1.420	large
$RR_{\mathrm{EOF}2}$	11°	13.161	[8.129, 20.000]	−1.082	large	5°	4.731	[3.674, 6.000]	−0.501	medium
$RR_{\mathrm{EOF}3}$	12°	12.000	[8.955, 15.292]	−1.503	large	14°	20.000	[17.511, 22.400]	−3.345	large

* Measured in $\frac{\mathrm{breaths}}{\min}$.

**Table 4:** Significance testing results using RMSE and MAE as comparison metrics on four estimators (populations) and 209 samples (paired videos).

	RMSE (Remote)					MAE (Remote)			
	R	MED *	CI	γ	Magnitude	MED *	CI	γ	Magnitude
$RR_{\mathrm{avg}}$	1°	4.108	[3.405, 4.947]	0.000	negligible	3.422	[2.756, 4.044]	0.000	negligible
$RR_{\mathrm{EOF}2}$	2°	6.074	[4.813, 6.992]	−0.751	medium	4.731	[3.765, 5.911]	−0.501	medium
$RR_{\mathrm{MTTS}\text{-}\mathrm{CAN}}$	3°	6.491	[5.449, 7.803]	−0.673	medium	4.711	[3.700, 6.529]	−0.375	small
$RR_{\mathrm{IMF}2}$	4°	7.578	[6.340, 9.242]	−1.128	large	6.933	[5.521, 8.333]	−1.117	large

* Measured in $\frac{\mathrm{breaths}}{\min}$.

## Data Availability

Data for reproducing the experiments reported in this article are available at this https://www.cs.binghamton.edu/~lijun/Research/3DFE/3DFE_Analysis.html (accessed on 20 March 2023).
